# Prior COVID‐19 infection among newly diagnosed tuberculosis patients in a tertiary care center in Tehran: A case‐control study

**DOI:** 10.1002/iid3.1275

**Published:** 2024-05-28

**Authors:** Kiavash Semnani, Marjan Sohrabi, Parvaneh Ebrahimi Alavijeh, SeyedAhmad SeyedAlinaghi, Shirin Esmaeili, Farzin Halabchi, Zahra Alizadeh, Amir Salami, Arezoo Salami Khaneshan

**Affiliations:** ^1^ Tehran University of Medical Sciences School of Medicine Tehran Iran; ^2^ Department of Infectious Diseases, Tehran University of Medical Sciences Imam Khomeini Hospital Complex, Tohid Squre Tehran Iran; ^3^ Department of Infectious Diseases, Tehran University of Medical Sciences Arash Women's Hospital Tehran Iran; ^4^ Iranian Research Center for HIV/AIDS, Tehran University of Medical Sciences Iranian Institute for Reduction of High Risk Behaviors Tehran Iran; ^5^ Department of Sports and Exercise Medicine Tehran University of Medical Sciences Tehran Iran; ^6^ Student Research Committee Iran University of Medical Sciences School of Medicine, Hemmat Hwy Tehran Iran

**Keywords:** corticosteroid, COVID‐19, prevention, pulmonary, reactivation, risk, Tocilizumab, tuberculosis

## Abstract

**Objective:**

To assess the risk of developing pulmonary tuberculosis (TB) in accordance with prior history of COVID‐19 infection.

**Background:**

Since the advent of the COVID‐19 pandemic much discussion has been had on the possible role it might play on global efforts to combat TB; most, focusing on the pandemic's impact on health care systems' capabilities to manage TB cases. Mechanisms have also been proposed by which the COVID‐19 infection may directly affect individuals' chance of developing TB infection. Cases have been reported with a history of COVID‐19 infection preceding a diagnosis of TB, evidencing its possible role as a risk factor for the disease.

**Methods:**

A case‐control study was conducted enrolling patients diagnosed with pulmonary TB in the absence of major risk factors previous history of TB, (HIV) human immunodeficiency virus infection), end‐stage renal disease, organ transplants, and use of immunosuppressive agents) for developing TB. Each patient was age and sex matched with one healthy control. Data regarding prior COVID‐19 infection, diabetes, and smoking status as well as the use of corticosteroids and Tocilizumab for the treatment of COVID‐19 infection was obtained. Bivariate analysis was conducted and variables with a likely association with TB status were entered in a multivariate model.

**Results:**

Bivariate analysis demonstrated a significant relationship between prior COVID‐19 infection and TB (95% confidence interval = 1.1−22.8, odds ratio [OR] = 5). Among other variables the severity of COVID‐19 infection was found to have a likely association with TB status (*p* = .125). In a multivariate model, prior COVID‐19 infection per se, was not found to be significantly associated with TB (*p* = .12, OR = 4.5).

**Conclusions:**

There seems to be an association between prior history of COVID‐19 and a future diagnosis of TB partially linked to the severity of disease. The findings of the current study may serve as a basis for further studies to determine the need for and efficacy of measures to follow‐up COVID‐19 patients at an increased risk for developing TB.

## Graphical abstract

COVID‐19 has been proposed to facilitate reactivation of pulmonary tuberculosis (TB) via immune‐dysregulation and local effects. The present study aimed to explore the possibility of such an association among patients diagnosed with TB using a case‐control design. Our findings suggest a link between prior history of COVID‐19 infection and the development of TB.

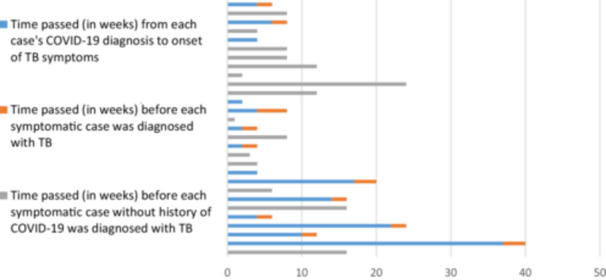

## 1 INTRODUCTION

COVID‐19 has affected 767 million people, and claimed more than 6.9 million lives.^1^ It seems fitting that this pandemic garnered unprecedented attention and impacted health care expenditure worldwide,^2^ but as we transition away from the pandemic, It is imperative that appropriate care be given to other infectious diseases with broad effect. Of these, Tuberculosis (TB) was the top cause of infectious deaths prepandemic with an estimated 10 million cases and 1.5 million death each year.^3^

TB is a mycobacterial infection spread mainly via respiratory droplets, and manifested in a variety of pulmonary (PTB) and extrapulmonary (EPTB) presentations.^4^ Among those infected with TB only 10% develop primary infection; with a remaining majority developing latent TB infection (LTBI).^5^ It is estimated that LTBI affects a third of the world's population, of whom 10% will progress to disease; half of these in the first 2 years of infection.^6^ These estimates are confounded by the lack a direct means of detection for live mycobacteria in LTBI.^7^ Currently guidelines recommend testing for LTBI in high risk individuals using a Tuberculin Skin Test or interferon‐gamma release assay.^8^

TB has maintained epidemiological significance in the pandemic era. Despite an initial 18% decrease in case notifications in 2020, partially attributable to disruptions in case detection and reporting, 7.5 million cases were reported in 2022—16% more than reports of 2020 and 28% more than 2021 and “the highest number for a single year” since the first WHO global TB report.^9^ Furthermore the trend of decreasing TB mortality had been reversed since 2020 with WHO estimating 1.6 million deaths attributable to TB in 2021 reflecting a possible uptake in undiagnosed or untreated TB^10^; an estimated death toll that later returned to the pre‐pandemic era estimations of 1.4 million deaths with an uptake in notified cases.^9^

Similarities between COVID‐19 and TB aren't limited to their infectious origin, and primary means of transmission.^11^ They also share an array of predominantly respiratory symptoms and predisposing comorbidities for example, diabetes mellitus (DM), chronic lung disease, and immunocompromised status.^12^ The population most affected by these two infections, also, seems to overlap^13^ taking the heaviest toll on those with a lower socioeconomic status.^14^ These commonalities have prompted several attempts at understanding the interplay between the two diseases.

Studies on the effect of prior respiratory epidemics on TB have found a rise in the number of TB cases ensuing the Spanish flu (1918) and reports of post‐SARS and MERS TB cases.^15^ Several cases of TB following COVID‐19 infection have been reported. 14 patients in a cohort of 49 with TB and COVID‐19 were diagnosed with TB following a diagnosis of COVID‐19.^16^ However, there was little delay between the diagnoses suggesting an underlying TB being brought to attention due to symptoms of COVID‐19. A similar study including 747 cases of TB‐COVID‐19 co‐infection from 34 countries found that in 71 of them COVID‐19 preceded TB, with half of these patients demonstrating radiographic evidence of old TB.^17^ Conversely among two cohorts of New York patients 63 out of 106 coinfected cases and 62 out of 133 cases with history of these infections more than 4 months apart were first diagnosed with COVID‐19.^18^ Other cases have demonstrated a more clear temporal relationship between the two infections but may be confounded by pre‐existing risk factors for TB.^19^, ^20^, ^21^, ^22^, ^23^ Njekwa et al. reported an interesting case of post‐COVID TB reactivation without predisposing factors.^24^ A systematic review of the literature found 33 patients diagnosed with TB up to 7 months after a diagnosis of COVID‐19.^25^

The possibility of a COVID‐19 infection influencing the future risk of TB development through hypothetical immunological mechanisms has remained elusive. The authors have sought to investigate the plausibility of such an occurrence via determining the prevalence of prior COVID‐19 infection in patients diagnosed with TB and further evaluating of the validity of such history as a risk factor for TB development in comparing cases and controls. This would serve as a basis for further enquiries into this matter and, if present, the establishment of the proposed influence of COVID‐19 infection as a risk factor for TB in population‐based studies.

## 2 METHODS

### 2.1 Study design and population

Due to the relative rarity and latency of TB diagnoses, a retrospective case‐control study was conducted. Cases included patient diagnosed with pulmonary TB in the Imam Khomeini Hospital Complex (IKHC), Tehran, Iran in the period of 1 year. Each case was matched for age and sex with one control from the outpatient sports medicine clinic of the same center.

The required sample size of 32 was calculated^26^ presuming a 35% prevalence of COVID‐19 in the study population^27^ and an odds ratio of five consistent with that of diseases with similar underlying pathophysiology.^28^, ^29^, ^30^

### 2.2 Inclusion and exclusion criteria

All adults (18 years of age or older) diagnosed with pulmonary TB during the study period were enrolled in the study. Diagnosis in each case was confirmed via microbiological (acid‐fast bacilli smear or culture) or molecular (MTB‐PCR or Xpert MTB/RIF) methods.^31^ Patients with a history of a major risk factors for developing TB were excluded. Conditions considered as major risk factors for TB included a prior history of TB, (HIV) human immunodeficiency virus infection, end‐stage renal disease and dialysis, organ transplant or malignancy, and the use of immunosuppressive medications (particularly corticosteroids and TNF inhibitors) for conditions other than COVID‐19.

### 2.3 Data collection

Patient data was retrieved using a telephone interview. Each patient was asked about their demographic data (age and sex), history of COVID‐19 diagnosis (confirmed by COVID‐19 PCR) before the ending of the study period, the severity of COVID‐19 infection, and immunosuppressive treatments received for this condition including corticosteroids and Tocilizumab. COVID‐19 infection was considered to be severe in the presence of SpO2 < 94 on room air, respiratory rate > 30 breaths/min, or lung infiltrates > 50% on their CT scan.^32^ There was also inquiry about a diagnosis of DM and smoking as two of the most common local risk factors for the development of TB.^33^ Patients were also asked about the presence of household exposures to TB.

Controls were interviewed in person. They were similarly asked to provide their age and sex as well as any history of PCR‐confirmed diagnosis of COVID‐19 before the termination of the study period, the severity of said diagnosis, and the use of immunosuppressive agents in the course of their treatment. Controls’ diabetes and smoking status was also recorded.

Patients and controls were asked to provide appropriate documentation of reported data‐points to minimize the possibility of recall bias. Nonresponders were excluded from the study.

### 2.4 Data analysis

Conditional logistic regression was used to determine the possible association between prior COVID‐19 infection and the development of TB. Bivariate analysis was conducted using the OpenEpi pair‐matched case‐control study tool to examine the relationship between each independent variable and TB status.^34^ Factors accounted for included prior COVID‐19 infection, a history of severe COVID‐19 infection, diabetes, smoking, and the use of corticosteroid and Tocilizumab in treatment of COVID‐19. Independent variables with a likely relationship to TB status (Exact *p* < .2) were then entered into a multivariate model to adjust the association between prior COVID‐19 and TB diagnosis. IBM SPSS statistics Cox regression tool was used to emulate this analysis for our matched data.^35^ In each case the odds ratio with its respective 95% confidence interval was calculated. Delay in TB diagnosis was compared between patients with and without exposure to COVID‐19 using an independent t‐test. Ancillary findings of latency from COVID‐19 infection to TB diagnosis, and household exposure to TB were reported without further analysis.

## 3 RESULTS

One hundred and forty‐four adults were diagnosed with pulmonary TB in the Imam Khomeini Hospital Complex during the study period. Patients were aged 18−96 years old with the average age of a patient being 51 years old. Ninety‐five patients were male (66%) and 49 were female (34%).

**Figure 1:**
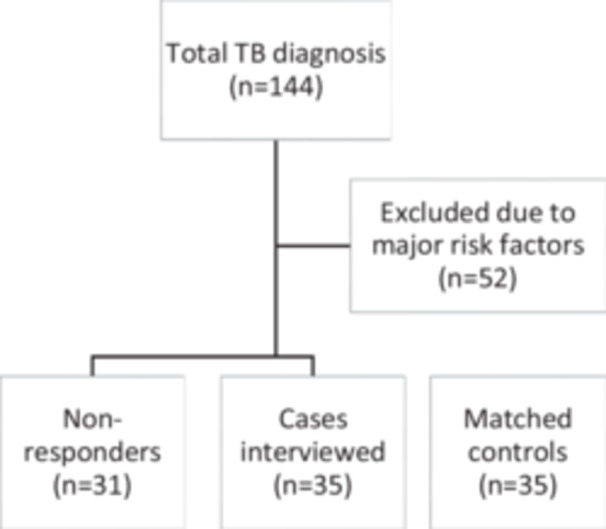
Flow of cases.

Fifty‐two patients were excluded due to the presence of major risk factors for developing TB. Eleven patients were excluded with a prior history of TB, 21 because of a positive HIV status, five due to a history of end‐stage renal disease and dialysis, five with organ transplant or known malignancy, and 10 for the use of immunosuppressive medications for conditions other than COVID‐19. Another 26 patients had expired before data gathering and thus were excluded. Out of the remaining 66 patient, the authors were able to establish a connection with 35 (Figure 1)—satisfying our calculated sample size.

In the final sample 21 (60%) and 14 (40%) patients were, respectively, male and female; a sex ratio which was comparable to all patients with this diagnosis (*p* = .22). Patients' age ranged from 19−78 years old with an average of 49 years old (*p* = .53). Seven patients (three male and four female) with an average age of 35 years old (*p* = .02, *d* = 1.05) reported a history of household exposure to TB. Upon review of medical documents no patient had reported pregnancy or breastfeeding. The average delay between onset of TB symptoms and diagnosis was 4.45 weeks; 1.5 weeks in patients with prior history of COVID‐19 infection and 7 weeks in those without such exposure (*p* = .003, *d* = 1.1).

Prior COVID‐19 infection was the only factor found to be significantly associated with a diagnosis of pulmonary TB (*p* = .038, OR = 5, Table 1). Out of the other variables analyzed only the severity of COVID‐19 infection was found to have a likely association with TB status. These two variables were entered into a multivariate model (Table 2). Adjusting for severe cases of COVID‐19 infection, prior history of COVID‐19 was not found to have a significant association with pulmonary TB (*p* = .12, OR = 4.5).

**Table 1:** Characteristics of cases and controls.

Independent variable	Cases *n* (%)	Controls *n* (%)	95% CI	OR	*p*
COVID‐19 infection	16 (45)	8 (22)	1.1−22.8	5	.038
Severe COVID‐19	7 (20)	2 (6)	0.7−49.8	6	.125
Diabetes	8 (22)	5 (14)	0.4−35.7	4	.375
Smoking	8 (22)	9 (25)	0.2−2.9	0.8	.999
Corticosteroid use^a^	4 (11)	1 (3)	0.4−35.7	4	.375
Tocilizumab use^a^	1 (3)	0 (0)	‐	‐	‐

Abbreviations: CI, confidence interval; OR, odds ratio.

a In treatment of COVID‐19 infection.

**Table 2:** Multivariate model considering variables with a likely association to TB.

Independent variable	95% confidence interval	Odds ratio	*p*
COVID‐19 infection	0.67−30.07	4.5	.120
Severe COVID‐19	0.15−9.40	1.2	.861

Abbreviation: TB, tuberculosis.

**Figure 2:**
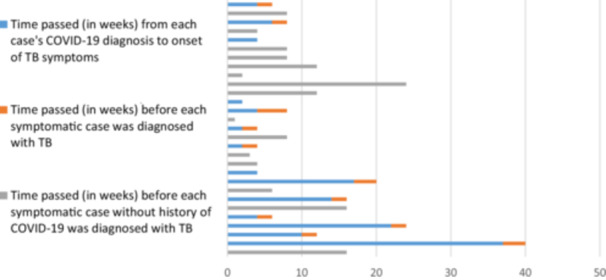
Delays in TB diagnosis for each case. TB, tuberculosis.

The average time between the two diagnoses was 10 weeks (Figure 2). Four out of sixteen patients was diagnosed with TB more than 3 months anteceding their COVID‐19 infection. Only one patient reported their COVID‐19 diagnosis to be more than 6 months before being diagnosed with TB.

Six of thirty‐five cases (17%) were diagnosed with COVID‐19 within the course of their TB diagnosis. Another three (8%) patients were diagnosed with TB (8−16 weeks) before COVID‐19. These cases were regarded as co‐infection rather than COVID‐19 associated TB and as such were considered to be negative for a prior history of COVID‐19 infection. Only two of these patients experienced severe COVID‐19.

## 4 DISCUSSION

The demographic profile of patients in the current study was found to be similar to that of previous studies. The TB/COVID‐19 Global Study Group had previously reported a cohort of 747 cases of TB‐COVID‐19 coinfection with an average age of 44 (31−58) years old; 70% of whom were male,^17^ comparable to our sample with an average age of 49 years old, 60% of whom were male. However the pattern of comorbidities in the study sample seems to deviate from that of previous reports. Srivastava and Jaggi^36^ reported 18 cases of post‐COVID‐19 TB of whom 50% had a history of diabetes, whereas we found 22% of our patients having such diagnosis. There also was a high prevalence of severe COVID‐19 cases in their sample with 50% patients having received corticosteroids and 22% having been treated using Tocilizumab while only 20% of our patients fulfilled the criteria for severe COVID‐19 infection and a mere 6% received Tocilizumab. Only 27% of their patients reported lacked a known risk factor for developing TB. Alemu et al.^25^ had a similar finding with 69% of their cases reporting comorbidities. In contrast 56% of the patients in the current study with a history of COVID‐19 had developed TB sans any major or prevalent predisposing factor for the disease.

Regarding the pathophysiology by which COVID‐19 may enhance the development and progression several mechanisms may be of prominence. It is well known that immune dysregulation contributes to the dissemination of active TB and reactivation of LTBI.^37^ One of the oft‐mentioned mechanisms pertains to the use of immunosuppressive treatments for COVID‐19. Use of corticosteroids is standard in severe cases of COVID‐19, and has shown to be a risk factor for TB reactivation.^13^ Although most studies have focused on long‐term treatment with corticosteroids in the context of rheumatologic disease,^13^ doses similar to those used for treatment of COVID‐19 have also been shown to increase the risk of TB.^38^ The WHO (2021, January 20; as cited in^39^) has also cautioned lack of proper LTBI case representation in corticosteroid trials for COVID‐19. Tocilizumab, another treatment for severe COVID‐19 cases, has also been linked with TB reactivation.^37^ With this, the authors were unable to detect a meaningful association between the use of these immunosuppressive treatments of COVID‐19 and later development of pulmonary TB.

Lymphopenia in the course of COVID‐19 has also been implicated as a possible contributing factor.^40^ Impairment in CD4+ T‐cell response facilitates TB dissemination by blocking granuloma formation.^41^ To this affect, a decrease in TB‐specific CD4+ response has been observed.^42^ Tangentially, researchers found a sixfold decrease in gamma interferon response among severe COVID‐19 patients regardless of lymphocyte counts or corticosteroid use status.^43^ Lung damage due to COVID‐19 constitutes another concern. Fibrosis caused by COVID‐19 may provide a basis for TB as interstitial lung diseases have been previously established as a risk factor for TB reactivation.^30^ Lastly coronavirus infection in a murine model has demonstrated promotion of a stem cell responses causing TB reactivation.^15^ Many of the above effects are tied to the severity of disease which seems to be in line with the findings of the current study.

An opposing explanation for the association between a prior diagnosis of COVID‐19 and a later diagnosis of TB is the extended testing efforts that may have resulted from heightened clinical sensitivity in the pandemic. Few of our patients even volunteered the history of a clinical misdiagnosis with COVID‐19 that was realized as TB with further paraclinical workup. Siranart et al.^44^ found most patients in their case‐series of 26 had been diagnosed with TB within 2 weeks of their COVID‐19 diagnosis lending further credence to this hypothesis. However, the current study found that there was a significant quantity of patients diagnosed with post‐COVID‐19 TB even excluding those diagnosed with TB in a continuum of clinical evaluation anteceding their confirmed diagnosis of COVID‐19.

Much has been explored about the ramifications of TB‐COVID‐19 co‐infection. The prevalence of such an occurrence was found to be minute (1.6%) among patients with COVID‐19.^45^ On the other hand among patients with TB the prevalence of co‐infection was a more notable 14%.^46^ This was even more common among TB patients in this study, with 25% having experienced co‐infection possibly due to a lesser regard for COVID‐19 preventive measures.

On other means by which the pandemic may have altered the course of TB development, it had been proposed that we may observe a shift in the mode of TB transmission towards household transmissions due to pandemic era social‐isolation.^47^ If true, this would necessitate targeted measures of infection control.^48^ We, however, were unable to observe such a phenomena with only 20% of TB patients reporting household exposure to TB. This number is similar to that found in studies from recent years before the COVID‐19 pandemic.^49^

## 5 CONCLUSIONS

This study found a statistically significant association (*p* = .038, OR = 5) between prior history of COVID‐19 infection and a future diagnosis with pulmonary TB suggesting its role as a possible risk factor or contributor to the development of TB. If expanded upon this finding may serve as an alarm for public health systems regarding the risk of an increasing TB load in the postpandemic era. We recommend prospective studies to better establish the nature of this association, and the validity of a targeted follow‐up or screening plan for TB in patients experiencing COVID‐19 focusing on cases with an enhanced risk for TB development (presence of other known major risk factors). Meanwhile it seems prudent to consider with greater care a diagnosis of TB in patients presenting with consistent symptoms and a recent history of COVID‐19.

### 5.1 Limitations

The most prominent limitation of this study is the restricted sample size and the resulting low power of analyses. This is apparent from the inability to detect an association between established risk factors for TB (diabetes and smoking) and its diagnosis in the current sample. We were also unable to conduct subgroup analysis comparing patients who underwent immune‐suppressive treatments to those without such a history due to the sparse number of patients fitting into the latter group. Further, the study design precludes us from establishing causality between the prior history of COVID‐19 and the development of TB.

The current study sample was selected among patients hospitalized in a tertiary care center and as such may entail the presence of confounding factors not accounted for. We were unable to control our results for the socioeconomic status and health habits of patients and controls. Mainly cultural sensitivities in interviews and sample size limitation prevented accounting for known risk factors such as TB contact, indoor air pollution, history of incarceration, and use of illicit substances in our final analysis. Another major risk factor unaccounted for was the prevalence of LTBI among cases before development of active TB as our means of data collection (medical documentations and patient interviews) seemed insufficient and unreliable to determine this information. Our study also presumes a standard prevalence of LTBI in the population as no data regarding LTBI testing was gathered nor any testing performed on the control group.

There may also be a degree of recall bias in acquiring diagnoses of COVID‐19 early in the pandemic when testing was yet to be prevalent in the study population. With these the authors uphold the need for large population‐based cohorts of COVID‐19 patients revisiting our findings.

## AUTHOR CONTRIBUTIONS

**Kiavash Semnani**: Conceptualization, data curation, formal analysis, investigation, methodology, visualization, writing—original draft. **Marjan Sohrabi**: Conceptualization, project administration, supervision, writing—review and editing. **Parvaneh Ebrahimi Alavijeh**: Project administration, supervision, writing—review and editing. **SeyedAhmad SeyedAlinaghi**: Formal analysis, methodology, verification, project administration, supervision, writing—review and editing. **Shirin Esmaeili**: Data curation, formal analysis, writing—original draft. **Farzin Halabchi**: Data curation, resources. **Zahra Alizadeh**: Data curation, resources. **Amir Salami**: Resources, validation, writing—review and editing. **Arezoo Salami Khaneshan**: Conceptualization, methodology, project administration, resources, supervision, writing—review and editing.

## CONFLICT OF INTERESTS STATEMENT

The authors declare no conflict of interest.

## ETHICS STATEMENT

The present study was approved by the Imam Khomeini Hospital Complex's ethics committee (IR.TUMS.IKHC.REC.1401.221) as part of a thesis in partial fulfillment of the requirements for the degree Doctor of Medicine. Verbal consent was acquired at the beginning of telephone interviews with cases and written consent was obtained from participants in the control group.

## Data Availability

Data supporting the findings of this study are available from the corresponding author upon reasonable request.
